# Prenatal diagnosis of Pallister‐Killian syndrome in one twin

**DOI:** 10.1002/ccr3.1624

**Published:** 2018-06-13

**Authors:** Lin Li, Linhuan Huang, Xuan Huang, Shaobin Lin, Zhiming He, Qun Fang

**Affiliations:** ^1^ Fetal Medicine Center Department of Obstetrics and Gynecology The First Affiliated Hospital of Sun Yat‐Sen University Guangzhou China

**Keywords:** dichorionic diamniotic twins, microarray analysis, Pallister‐Killian syndrome, prenatal diagnosis

## Abstract

Pallister‐Killian syndrome (PKS) is often incidentally diagnosed prenatally due to ultrasound abnormalities or advanced maternal age. Severely shortened limbs could be the most outstanding abnormal observation in a fetus with PKS. PKS can be detected with the highest mosaic ratio by chromosomal microarray analysis (CMA) on uncultured amniocytes prenatally.

## 1 INTRODUCTION

Pallister‐Killian syndrome (PKS, OMIM #601803) is a rare, sporadic genetic disorder caused by the presence of extra copies of the short arm of chromosome 12, which most commonly exists as a supernumerary isochromosome, 12p. Manifestations of PKS potentially involve many systemic abnormalities, including seizures, intellectual impairment, hearing loss, congenital heart defects, intestinal malrotation, pigmentary skin anomalies, and a variety of congenital malformations.^1^ However, there is a wide range of variability in phenotypic expression, ranging from intrauterine death to very mild forms.^2^

Pallister‐Killian syndrome is characterized by tissue‐limited or tissue‐specific mosaic tetrasomy of 12p.^3^ The mosaic ratio of tetrasomy cells does not correlate with the severity of congenital abnormalities,^4^, ^5^ which makes prenatal diagnosis and genetic counseling difficult. Here, we report a case of Pallister‐Killian syndrome, diagnosed by chromosomal microarray analysis (CMA), in one fetus of a pair of dichorionic diamniotic twins. Abnormalities including a thickened nuchal fold, short limbs, and mild lateral ventriculomegaly were revealed by ultrasound. The mosaic supernumerary isochromosome 12p was confirmed prenatally by karyotype and metaphase fluorescence in situ hybridization (FISH) analysis on cultured amniocytes and blood lymphocytes.

## 2 CASE REPORT

**Figure 1:**
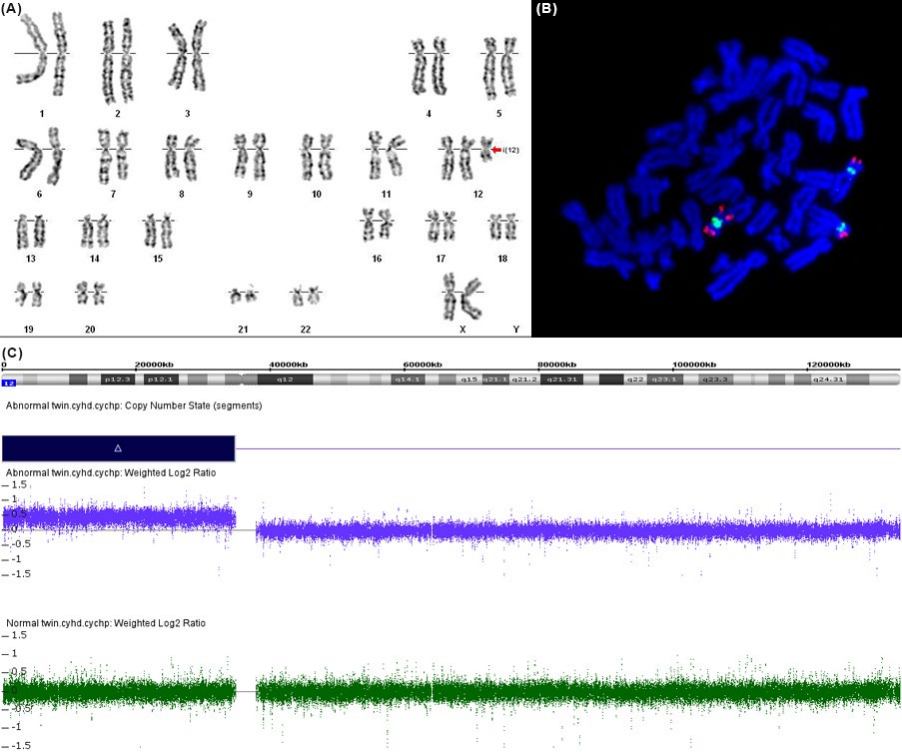
A, Amniocytes karyotype in the abnormal fetus showing a supernumerary isochromosome 12p (red arrow). B, Metaphase FISH showing an additional isochromosome 12p (a target probe at 12p13.33 labeled in red, and a control probe at the 12 centromere labeled in green) in the abnormal fetus. C, Chromosomal microarray analysis in the twins. Blue rectangle and the deviation of probe log2 ratios from 0 indicated the copy‐number gain on the short arm of chromosome 12, while the result of the normal fetus is shown in the green bar

A 34‐year‐old Chinese woman, gravida 1, para 0, was referred to our hospital for thickened nuchal translucency (3.4 mm) in one fetus of dichorionic diamniotic twins at 13 weeks of pregnancy. She underwent in vitro fertilization and embryo transfer (IVF‐ET) because her husband was oligoasthenospermia. Two embryos were transferred to the uterus. Transvaginal ultrasound revealed an unremarkable dichorionic twin pregnancy in the first trimester. Noninvasive prenatal testing (NIPT) was performed at 15 weeks of gestation, showing a low‐risk for fetal 21, 13 and 18 trisomy. After informed consent was obtained, she underwent amniocentesis for further molecular analysis at 17 weeks of gestation. The results of CMA showed a gain of the entire short arm of chromosome 12 in approximately 80% of cells in the fetus with thickened nuchal translucency, while normal in the other fetus (Figure 1E). SNP array analysis confirmed that the twins were dizygotic. Then, a second amniocentesis was offered to confirm the tetrasomy using FISH and G‐banding karyotyping at 20 weeks of gestation. The karyotyping showed that the abnormal fetus was 47,XX,i(12p)[40]/46,XX[10] (Figure 1A). FISH analysis confirmed tetrasomy 12p in 80% (20/25) of cells (Figure 1B). Both the karyotype and FISH results of the other fetus showed a normal female (Figure 1C,D).

The decision to terminate the abnormal fetus was difficult for the parents due to the wide spectrum of PKS manifestations. At 20 weeks of gestation, more abnormalities, including severely shortened humerus and femur (<−6 SD) and mild lateral ventriculomegaly, were revealed by three‐dimensional ultrasound. Meanwhile, head circumference, abdominal circumference, and biparietal diameter were in the normal ranges. In consideration of the ultrasonic and cytogenetic findings, the parents opted for selective termination. Transabdominal intrathoracic injection of potassium chloride (KCl) into the heart of the fetus with 12p tetrasomy was performed successfully at 23 weeks of gestation. Before the injection of KCl, heart blood was obtained and the karyotype analysis of heart blood in the abnormal fetus showed 47,XX,i(12p)[12]/46,XX[23]. Subsequently, ultrasounds were performed regularly, and the remaining fetus showed normal biometric parameters. A healthy female baby was born by normal vaginal delivery at term.

## 3 DISCUSSION

This case was diagnosed prenatally due to a thickened nuchal fold in one fetus of dichorionic diamniotic twins. Additional abnormalities, including short limbs and mild lateral ventriculomegaly, were revealed in the three‐dimension ultrasound. In the reported literature, other distinctive ultrasound findings in fetuses with PKS include a flat nasal bridge, hydrops fetalis, structural heart defects, diaphragmatic hernia, rhizomelic limb shortening, polydactyly and polyhydramnios.^6^, ^7^ Currently, most abnormal ultrasound findings associated with PKS can be detected prenatally. However, it remains problematic to choose the most appropriate tissue and technique for examination.

The mosaic ratio of the isochromosome 12p is variable among different tissues in an individual. The most appropriate specimen used to diagnose individuals with suspected PKS is postnatal skin fibroblasts.^2^ Stem cells containing this small marker chromosome remain relatively stable in skin fibroblasts, while they are prone to loss in continually dividing blood lymphocytes.^8^ The majority of cells present in the amniotic fluid are epithelial cells sloughing off from fetal skin. Kucińska‐Chahwan et al^9^ concluded that the percentage of aneuploid cells ranged from 23% to 100% in cultured amniotic fluid and from 0% to 23% in cultured cord blood in a retrospective analysis. Although the mosaic level is relatively higher in chorionic villi cells, confined placental mosaicism will lead to a possible false negative result when isochromosome 12p is present in the fetus but not in the placenta or the degree of mosaicism is much lower in the chorion villus.^6^ Chih‐Ping Chen et al suggested that more than one tissue should be used for confirmation because there was no tissue avoids a false negative result.^3^ In our case, the result showed that the mosaic ratio of blood lymphocytes was much lower than amniocytes (Table 1), which was in accordance with those cases reported in the literature.

**Table 1:** Comparison of the mosaic ratios of isochromosome 12p detected in different specimens by different techniques in the abnormal fetus

Gestational age (wk)	Specimens	Techniques	Mosaic ratios (%)
17	Amniocytes	CMA	80
20	Amniocytes	Karyotype	80
20	Amniocytes	FISH	80
23	Blood lymphocytes	Karyotype	34.3

CMA, chromosomal microarray analysis; FISH, fluorescence in situ hybridization.

The frequency of abnormal markers is extremely low in phytohemagglutinin (PHA)‐stimulated blood lymphocytes with standard karyotyping analysis, because abnormal cells may be less competitive than normal cells in the process of cell culturing.^4^, ^10^ CMA has an advantage in detecting low levels of mosaicism using genomic DNA isolated from an uncultured specimen.^4^, ^10^ However, there is also a risk of obtaining a false negative result if the ratio of isochromosome is low (<5%) in the tissue examined.^11^ In our case, the mosaic ratio of isochromosome 12p detected by CMA on uncultured amniocytes was the same as the mosaic ratio detected by karyotype and FISH on cultured amniocytes. We attributed the results to the increasing fetal epithelial cells in the amniotic fluid consistent with the increase in gestational age as keratinization of fetal skin mainly begins at 19‐20 weeks of gestation.^12^

The mechanism of isochromosome 12p formation is not fully understood. It is mostly caused by maternal meiosis II nondisjunction, generating a disomic gamete and then isochromosome formation postzygotically from one chromosome 12.^7^, ^10^ As with other aneuploidies, such as Down syndrome, advanced maternal age is a contributing risk factor for tetrasomy 12p.^8^ In the majority of cases, PKS is incidentally diagnosed prenatally due to advanced maternal age or ultrasound abnormalities.^13^ Maternal serum biochemistry shows an increased risk for Down syndrome in some cases.^6^, ^7^ Theoretically, there is no recurrence risk, unless germline mosaic isochromosome 12p is present in one of the parents. Parental cytogenetic analysis should be performed to rule out the balanced translocation of chromosome 12, which may cause the PKS phenotype in the fetus due to inherited unbalanced duplications on 12p.^1^

The parents described in this report chose selective feticide because of the genetic and congenital structural abnormalities, especially the severely shortened limbs. It is commonly considered that PKS is usually a severe disorder and termination is justified. Individuals affected by major structural anomalies, such as severe diaphragmatic hernia, hydrocephalus, and hydrops fetalis, would die in utero or postnatally.^5^ Although most individuals with PKS manifest with profound intellectual disability and sensory impairments, there are individuals who have only mild to moderate intellectual disability and attend regular schools.^14^ Kostanecka et al speculated that the underlying mechanism of such a wide phenotypic spectrum could be related to the differential expression of supernumerary 12p or differential effects on the cellular processes involved in brain development, but it is unrealistic to estimate the level of mosaicism in the dominant organs.

Currently, we can predict the manifestations according to the duplication region and genes included in the region by CMA. Izumi et al defined a critical region based on a patient with features similar to PKS caused by microduplications on 12p13.31, and the 2.8 Mb region containing 26 genes may be responsible for the core phenotype of PKS.^15^ Among these 26 genes, *ING4*,* CHD4,* and *ATN1* are the developmentally critical genes that result in the PKS phenotype. Other genes that may contribute to the clinical phenotype of *PKS* include *ZFPM2*,* GATA6*,* IGFBP2*,* NCAPD2*,* HOX,* and *SOX9*.^1^, ^2^ These genes may cause multisystem disorders of PKS, such as cognitive impairment, growth deceleration, congenital heart defects, diaphragmatic hernia, limb shortening, micrognathia and cleft palate. Although CMA allows us to speculate the possible prognosis according to the affected regions and genes, the highly variable features of PKS remain problematic for prenatal consulting. Further studies and advanced techniques are needed to be established the correlation between mosaic ratio and clinical manifestations. This case emphasizes that PKS can be detected with the highest mosaic ratio by CMA on uncultured amniocytes and both the results of prenatal diagnosis and ultrasound findings are necessary for genetic consulting.

## CONFLICT OF INTEREST

None declared.

## AUTHORSHIP

LL: collected clinical data and wrote the manuscript. LH, XH, and ZH: contributed to patient evaluation, diagnosis, treatment, and follow‐up. SL: performed genetic testing and provided technical support. QF: supervised and reviewed the manuscript.

## References

[1] Izumi K , Krantz ID . Pallister‐Killian syndrome. Am J Med Genet C Semin Med Genet. 2014;166C:406‐413. 25425112 10.1002/ajmg.c.31423

[2] Kaur M , Izumi K , Wilkens AB , et al. Genome‐wide expression analysis in fibroblast cell lines from probands with Pallister Killian syndrome. PLoS ONE. 2014;9:e108853. 25329894 10.1371/journal.pone.0108853 PMC4199614

[3] Chen C , Tsai F , Chern S , Lee C , Town D , Wang W . Cytogenetic variability in the proportion of abnormal cells between the various tissues in prenatally detected mosaic tetrasomy 12p. Prenat Diagn. 2007;27:1170‐1173. 17880035 10.1002/pd.1850

[4] Theisen A , Rosenfeld JA , Farrell SA , et al. aCGH detects partial tetrasomy of 12p in blood from Pallister‐Killian syndrome cases without invasive skin biopsy. Am J Med Genet A. 2009;149A:914‐918. 19353629 10.1002/ajmg.a.32767

[5] Schinzel A . Tetrasomy 12p (Pallister‐Killian syndrome). J Med Genet. 1991;28:122‐125. 2002482 10.1136/jmg.28.2.122 PMC1016781

[6] Langford K , Hodgson S , Seller M , Maxwell D . Pallister‐Killian syndrome presenting through nuchal translucency screening for trisomy 21. Prenat Diagn. 2000;20:670‐672. 10951480 10.1002/1097-0223(200008)20:8<670::aid-pd885>3.0.co;2-u

[7] Srinivasan A , Wright D . Pallister‐Killian syndrome. Am J Case Rep. 2014;15:194‐198. 24826207 10.12659/AJCR.890614 PMC4018245

[8] Wenger SL , Steele MW , Yu WD . Risk effect of maternal age in Pallister i(12p) syndrome. Clin Genet. 1988;34:181‐184. 3180504 10.1111/j.1399-0004.1988.tb02860.x

[9] Kucinska‐Chahwan A , Bijok J , Dabkowska S , et al. Targeted prenatal diagnosis of Pallister‐Killian syndrome. Prenat Diagn. 2017;37:446‐452. 28233318 10.1002/pd.5030

[10] Conlin LK , Kaur M , Izumi K , et al. Utility of SNP arrays in detecting, quantifying, and determining meiotic origin of tetrasomy 12p in blood from individuals with Pallister‐Killian syndrome. Am J Med Genet A. 2012;158A:3046‐3053. 23169773 10.1002/ajmg.a.35726

[11] Hodge JC , Hulshizer RL , Seger P , St AA , Bair J , Kirmani S . Array CGH on unstimulated blood does not detect all cases of Pallister‐Killian syndrome: a skin biopsy should remain the diagnostic gold standard. Am J Med Genet A. 2012;158A:669‐673. 22315202 10.1002/ajmg.a.35209

[12] Underwood MA , Gilbert WM , Sherman MP . Amniotic fluid: not just fetal urine anymore. J Perinatol. 2005;25:341‐348. 15861199 10.1038/sj.jp.7211290

[13] Doray B , Girard‐Lemaire F , Gasser B , et al. Pallister‐Killian syndrome: difficulties of prenatal diagnosis. Prenat Diagn. 2002;22:470‐477. 12116305 10.1002/pd.342

[14] Kostanecka A , Close LB , Izumi K , Krantz ID , Pipan M . Developmental and behavioral characteristics of individuals with Pallister‐Killian syndrome. Am J Med Genet A. 2012;158A:3018‐3025. 23169763 10.1002/ajmg.a.35670

[15] Izumi K , Conlin LK , Berrodin D , et al. Duplication 12p and Pallister‐Killian syndrome: a case report and review of the literature toward defining a Pallister‐Killian syndrome minimal critical region. Am J Med Genet A. 2012;158A:3033‐3045. 23169682 10.1002/ajmg.a.35500

